# Resveratrol-modified mesoporous silica nanoparticle for tumor-targeted therapy of gastric cancer

**DOI:** 10.1080/21655979.2021.1971507

**Published:** 2021-09-10

**Authors:** Mingzhen Lin, Wenxia Yao, Yao Xiao, Zhijie Dong, Wei Huang, Fan Zhang, Xinke Zhou, Min Liang

**Affiliations:** a Department of Oncology, The Fifth Affiliated Hospital of Guangzhou Medical University, Guangzhou Medical University, Guangzhou, China; b Department of Center Laboratory, The Fifth Affiliated Hospital of Guangzhou Medical University, Guangzhou Medical University, Guangzhou, China; c Department of Gastrointestinal Surgery, The Fifth Affiliated Hospital of Guangzhou Medical University, Guangzhou Medical University, Guangzhou, China

**Keywords:** Resveratrol, mesoporous silica nanoparticles, gastric cancer, therapy, biosafety

## Abstract

Resveratrol (Res) has been shown to exhibit anti-cancer properties in gastric cancer. However, its clinical application is limited by its poor pharmacokinetics, stability, and low solubility. Hence, this study aimed to explore and verify a better delivery system for gastric cancer therapy. Using transmission electron microscopy, Fourier transform infrared (FTIR) spectroscopy, and ultraviolet (UV) spectrometry, we observed the shape and encapsulation of resveratrol-modified mesoporous silica nanoparticles (MSN-Res) that were synthesized by chemical methods. To explore the anti-cancer effects of these MSN-Res in vivo and in vitro, we established AGS and HGC-27 tumor-bearing mouse models. Meanwhile, the proliferation of gastric cancer cells in vitro and in vivo was assessed by Cell Counting Kit-8, EdU, and Ki-67 immunohistochemical staining methods, while cellular apoptosis, and invasion and migration were detected by TdT-mediated dUTP nick end labeling (TUNEL) and Transwell assays, respectively. FTIR and UV results showed that we successfully synthesized and loaded drugs. Safety evaluation experiments showed that neither MSN-SH nor MSN-Res had toxic effects on the normal tissues of animals. Moreover, in vitro experiments revealed that MSN-Res significantly inhibited the proliferation, invasion, and migration of gastric cancer cells. Furthermore, TUNEL assay showed that MSN-Res promoted apoptosis in gastric cancer. These results were confirmed by the nude mouse tumorigenesis experiment. In conclusion, we demonstrated that MSN-Res showed better inhibitory effect on the development of gastric cancer than Res alone, indicating that MSN-Res could be a promising drug delivery system for gastric cancer treatment.

## 1. Introduction

Gastric cancer, the fourth most common cancer in the world, ranks second among all tumors in terms of fatality rate [1], which is associated with its high incidence of metastasis and recurrence [2]. At present, the main treatment methods for gastric cancer are surgery and adjuvant therapy, such as radiotherapy and chemotherapy [3]. However, gastric cancer is not sensitive to radiotherapy. Aside from this, the commonly used chemotherapy drugs, such as 5-FU, cisplatin, paclitaxel, and oxaliplatin, may have toxic side effects to patients [4]. Moreover, alterations in certain signaling pathways can result in gastric cancer cells developing chemotherapy resistance [5]. Hence, it is important to find new treatment methods that are highly effective but have reduced side effects.

Resveratrol (Res; 3,4,5-trihydroxystilbene) is a natural polyphenol found in many plants, such as grapes and peanuts [6]. Studies have shown that in addition to delaying aging, reducing oxidative stress, and preventing cardiovascular diseases, resveratrol can inhibit the occurrence and development of various cancers [7–9], including gastric cancer [10,11]. Ren et al. demonstrated that resveratrol induces endoplasmic reticulum stress-mediated apoptosis and G2/M phase arrest in AGS gastric cancer cells synergized with cisplatin [12]. Moreover, Kim et al. found that resveratrol suppresses gastric cancer cell proliferation and survival by inhibiting PIM-1 kinase activity [13]. These results indicate that resveratrol has promising application prospects in anti-cancer adjuvant drugs. However, the application of resveratrol is limited by factors, such as water solubility and low bioavailability [8]. Therefore, it is important to establish a more efficient delivery system that could also improve tumor drug targeting and enhance the bioavailability of resveratrol.

In recent years, drug carriers have become a research hotspot in the field of biomedicine, with most studies aiming to reduce the side effects of drugs, and to improve the distribution and metabolism of drugs in the body, and the efficiency of the treatment. Nanoparticles are now favored by researchers because of their high surface area-to-volume ratio and high reactivity, and are expected to have increased application [14]. Among them, mesoporous silica nanoparticles (MSNs) have attracted widespread attention because of their ordered mesoporous structure, large specific surface area, good biocompatibility, and high drug loading rate. Moreover, they are considered to be one of the most promising drug carriers available [15–17]. Several studies have also shown that MSN can act as an effective drug carrier in gastric cancer. Fang et al. indicated that quercetin and doxorubicin co-delivery using MSN enhanced the efficacy of gastric carcinoma chemotherapy [18]. Hu et al. developed anti-miR21 and Res-loaded MSNs conjugated with hyaluronic acid (HA), which enhanced the therapeutic efficacy in gastric carcinoma [19]. In their study, there was a synergistic antitumor effect of the combined use of the anti-miR21 and Res, whereas our study not only aimed to investigate the antitumor effect in the other two kinds of mice, but also verified the biosafety of the application of Res in vivo. These results provide theoretical basis for the use of res-loaded MSNs in the treatment of gastric cancer.

We hypothesized that MSNs could be beneficial in the treatment of gastric cancer with the promising drug Res. Thus, in the present study, we aimed to develop Res-loaded MSNs and to assess their therapeutic efficacy in the treatment of gastric cancer, in terms of water solubility and bioavailability.

## 2. Materials and methods

### 2.1 Preparation and surface modification of MSNs

Commercialized MSNs were purchased from Xi’an Ruixi Biological Technology Co., Ltd (Shanxi, China). The surface of MSNs was modified successively with functional groups -SH and – -SH-COOH, and then Res was loaded onto the surface of modified MSNs and modified by PEI-FA, following the methods by [20]. Briefly, 0.84 g cetyltrimethylammonium bromide (CTAB) and 2 M NaOH (2.8 mL) were added to 400 ml of deionized water, and then heated to 80°C. After which, 4.8 mL of tetraethyl orthosilicate was slowly added to the solution. After 10 min, 0.792 mL of 3-mercaptopropyltrimethoxysilane (MPTMS) was added, and the mixture was kept at 80°C for 2 h. The resulting MSN-SH was washed with distilled water and ethanol three times, and then dried under vacuum for 12 h. Then, MSN-SH-COOH was obtained by allowing MSN-SH to react with 0.5 g 2,2-Dipyridyl disulfide (Py-SS-Py), 12.5 mL dimethylformamide (DMF), 0.36 mL 3-mercaptopropionic acid and 0.5 ml of acetic acid for 24 h at 40°C.

### 2.2 Res loading onto surface of modified MSNs

Modified MSN MSN-SH-COOH (20 mg) was resuspended in 1.5 mL of absolute ethanol containing 6 mg of Res by ultrasound, and the mixture was stirred in the dark for 24 h. The resulting RES-loaded MSNs were centrifuged and washed with ethanol and water before freeze-drying. The MSN of RES was conjugated with PEI-FA to obtain MSN/Res-PEI-FA. Briefly, 30 mg of FA was dissolved in dimethyl sulfoxide (DMSO) and 1-ethyl-3-carbodiimide hydrochloride (6 mg/mL) and N-Hydroxysuccinimide (3 mg/mL) were added to activate the carboxyl group. Subsequently, 7 mL DMSO that contained 300 mg PEI was added, then mixture was stirred for 24 h. After this, excess FA was removed by adding 10 ml ultrapure water, and then the mixture was centrifuged, purified, and freeze-dried to obtain MSN/Res-PEI-FA.

### 2.3 Characterization of Res-loaded MSN

The sub-localization of Res-modified MSNs was observed using transmission electron microscopy (TEM). Briefly, Res-loaded MSNs were fixed with 2.5% glutaraldehyde solution for 2 h and then rinsed with 0.1 M precooled phosphate-buffered saline (PBS; pH 7.0). After which, 2% osmium tetroxide (OsO_4_) and 0.1 M cacodylate buffer (pH 7.4) were used to post-fix cells for 2–3 min and then dried at room temperature. The cells were observed and imaged using TEM (HT7700; Hitachi, Tokyo, Japan) at 80 kV by two blinded pathologists. Fourier transform infrared (FTIR) spectroscopy using an FTIR spectrometer (PerkinElmer, Inc., Waltham, MA, USA) and UV spectrophotometry was performed to confirm the incorporation and absorption peaks of MSNs with different functional groups and Res. Cytotoxicity assay of Res-loaded MSN was performed by cell proliferation test, hemolysis rate detection, and organ damage detection by hematoxylin and eosin (H&E) staining.

### 2.4 Cell culture and treatment

Human gastric cancer cell lines HGC-27 and AGS were purchased from the Type Culture Collection of the Chinese Academy of Science (Shanghai, China). After cell line recovery, cells were cultured in 90% Dulbecco’s modified Eagle’s medium (DMEM; 12,430,054, Gibco, Thermo Fisher, Waltham, MA, USA) supplemented with 10% fetal bovine serum (FBS, Gibco), 100 U/mL penicillin, and 100 μg/mL streptomycin at 37°C in a 5% CO_2_-contained incubator under 95% saturation humidity. HGC-27 and AGS cells were seeded in a 6-well plate which was treated with MSN, Res, or Res-loaded MSNs with different concentrations until 70–90% confluence was reached.

### 2.5 Cell Counting Kit-8 (CCK8) and EdU assay

Cell proliferation was assessed using the CCK8 assay kit (CK04, Dojindo Molecular Technologies, Inc, Rockville, MD, USA) and EdU dye solution (C10327, Guangzhou RiboBio Co., Ltd, Guangzhou, China) according to the manufacturer’s instructions. For the CCK8 assay, 1 × 10^4^ HGC-27 and AGS cells were incubated in a 96-well plate, then treated with MSN, Res, and Res-loaded MSNs with different concentrations when 70–90% cell confluence was reached. The cells were harvested, washed, and resuspended in 100 μL fresh complete medium at 0, 24, 48, and 72 h after incubation. Subsequently, the cells were incubated with 10 μL CCK8 solution for 4 h, then the absorbance (OD) at 450 nm was determined using a microplate reader.

For the EdU assay, the cells treated with MSN, Res, and Res-loaded MSNs were harvested and washed with PBS. EdU solution (150 μL, reagent A, 50 μM) was added to each well, then the plate was incubated for 2 h. After which, the cells were fixed with 200 μL 4% paraformaldehyde at room temperature overnight, then stained with DAPI for 30 min in the dark at room temperature. Finally, the cells were washed, and images of different fields were taken using a fluorescence microscope (Olympus, Tokyo, Japan) with CY3 at 590 nm.

### 2.6 Migration and invasion detected by transwell assay

Cell invasion and migration abilities of the HGC-27 and AGS cells were evaluated using the transwell assay [21], which was performed using Millipore transwell chambers (8 μm pore size, Merck Millipore, Burlington, MA, USA). HGC-27 and AGS cells (2 × 10^4^) in each well were treated with MSNs, Res and Res-loaded MSNs, and then seeded in the upper chambers of the 12-well plate (Corning, Tewksbury, MA, USA) in 500 μL serum-free medium, and the lower chambers were filled with complete medium (1 mL), then incubated for 24 h at 37℃. The cells on the upper surface of the membrane and in the lower chamber were removed with a cotton swab, fixed with methanol, and stained with Giemsa (HiMedia Labs, Mumbai, India) at the end of incubation. Images from different fields were captured with an inverted microscope (CX41, Olympus). The cell invasion assay was almost the same as the transwell migration assay, except that the transwell chambers were coated with 1 mg/ml growth factor-reduced Matrigel (Corning) prior to the assay.

### 2.7 TUNEL assay for cell apoptosis

The apoptosis of HGC-27 and AGS cells in vitro and in vivo was detected by TdT-mediated dUTP nick end labeling (TUNEL) assay (KGA7061, KeyGen, Nanjing, China), following the manufacturer’s instructions. For in vitro detection, HGC-27 and AGS cells were washed twice with PBS and immobilized with 4% paraformaldehyde. Meanwhile, the tissue specimens were sliced into 4 μm sections then embedded in paraffin. Subsequently, the slices were treated with Proteinase K for 30 min at 37°C. After rinsing, they were incubated in the TUNEL reaction mixture at 37°C for 60 min, then washed three times with PBS. The slices were then incubated in 150 μL Hoechst 33,258 for 20 min in a dark room. Images were taken using an immunofluorescence microscope (IX71, Leica, Wetzlar, Germany), and the number of apoptotic cells was determined using Image-Pro Plus 6.0.

### 2.8 Xenograft tumor experiment

Male Balb/c nude mice weighing 20–25 g at 3–4 weeks were purchased from the Animal Experiment Center of Southern Medical University (Guangzhou, China). The nude mice were kept under a 12:12-h light-dark cycle in an SPF animal laboratory, with 60–65% humidity at 22–25℃. For the establishment of tumor-bearing mice models, the nude mice were randomly divided into four groups (n = 5 per group): control, Res, MSNs, and MSN/Res groups. A total of 3 × 10^6^ cells were injected subcutaneously into the flanks of nude mice to generate a xenograft tumor model after treatment with Res, MSNs, and MSN/Res. The size of the tumor (V = A long diameter×B2 short diameter/2) was observed and recorded 28 d after injection. All experiments were approved by the Ethics Committee of the Southern Medical University.

### 2.9 Hematoxylin and eosin staining and Ki67 immunohistochemistry

The tumor tissues were obtained, dissected, fixed in 10% buffered formalin, and processed in a paraffin tissue processing machine from different groups. Then, the tumor was cut into 7-μm sections, and stained with H&E (G1005, Servicebio, China). Ki67 immunohistochemistry was used to detect the proliferation of Ki67 HGC-27 and AGS cells in the tumor tissue. Briefly, the sections were inactivated in 3% catalase for 15 min in an oven for 1 h at 60°C. Then, the sections were blocked with 5% bovine serum albumin (BSA) for 30 min at 37°C. Subsequently, the sections were incubated with KI67 rabbit polyclonal antibody (1:500; 27,309-1-AP, Proteintech, China) at 4°C overnight, followed by incubation with biotinylated goat anti-rabbit IgG (1:1000; ab6721, Abcam, Cambridge, UK) at 37°C for 30 min. Color rendering was performed using DAB (K5007, DAKO), and the sections were kept at room temperature for 8 min in a dark room. Finally, images were captured using an XSP-36 microscope (Boshida Optical Co., Ltd, Shenzhen, China).

### 2.10 Statistical Analysis

Data in the present study were analyzed using SPSS (version 21.0; SPSS, Inc., Chicago, IL, USA) software and are presented as means ± standard deviation (SD). All experiments were done with at least three replicates. Student’s t-test and one-way analysis of variance (ANOVA) followed by Dunnett’s multiple comparison test were used to calculate the statistical significance between two and more than two groups. Statistical significance was set at P < 0.05.

## 3. Results

In the current study, we hypothesized that Res-loaded MSNs can serve as an efficient Res delivery system and can be applied in the treatment of gastric cancer. To determine the possibility of delivery system construction, biosafety, and antitumor effects, a series of experiments were performed. Finally, we confirmed that the Res-loaded MSNs exhibited a suppressive effect on gastric cancer in vitro and in vivo with high biosafety.

### 3.1 Preparation and characterization of Res-loaded MSN

**Figure 1.**
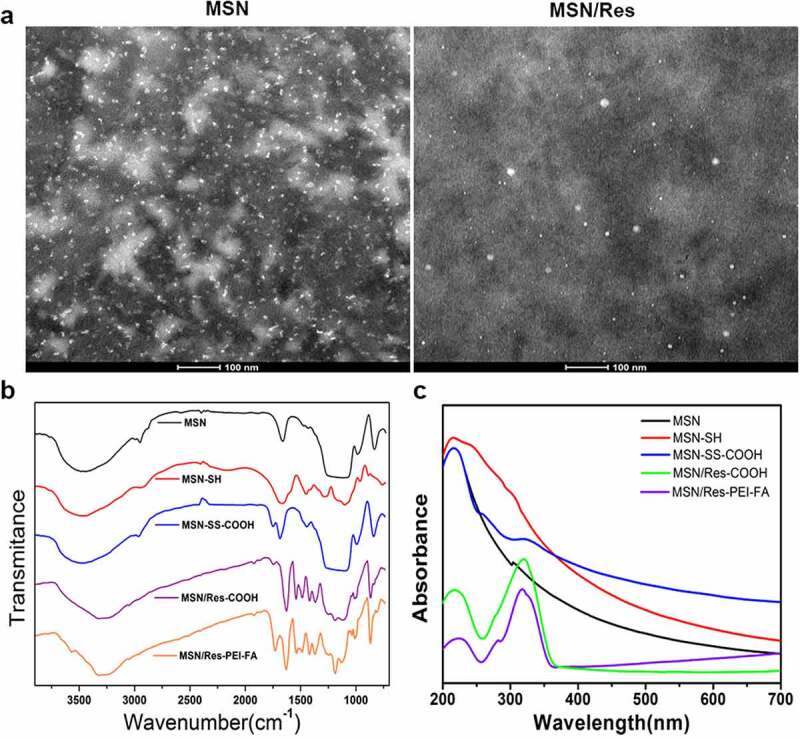
Preparation and characterization of resveratrol-loaded (Res-loaded) mesoporous silica nanoparticles (MSN)

As shown in Figure 1a, MSN and MSN/Res were homogeneous, with diameters within 100 nm. TEM results revealed that MSN and MSN/Res were composed of spheroid nanoparticles. FTIR spectroscopy indicated that the pristine MSNs presented peaks at 1,080 cm-1 and 810 cm-1, which corresponded to Si-O and Si-O-Si vibrations of silanol groups, and the different bonds and hydroxyl groups on the surface of MSNs. The methylene stretching vibration peak at 2926 cm-1 and the flexural vibration peak of amino group of 1470 cm-1 both indicate the success of the amination modification process. Moreover, the vibration peaks of carboxyl occurred at 1810 cm-1 for MSN-SS-COOH, MSN/Res-COOH, and MSN/Res-PEI-FA. In addition, the vibration peaks of Res and the benzene ring skeleton were observed at 1500 cm-1 (Figure 1b). These results demonstrated that the different functional groups were successfully modified on the surface of MSNs, and that Res was successfully encapsulated. This was also supported by the UV spectrum of MSN-SS-COOH, MSN/Res-COOH and MSN/Res-FEI-FA, which showed a small peak at 325 nm, indicating the successful encapsulation of Res. (Figure 1c).

A, Transmission electron microscopy (TEM) was used to detect the effect and sub-localization of MSNs and Res-loaded MSN to gastric cancer cells; Scale bar = 100 μm; B, Fourier transform infrared (FTIR) spectroscopy was used to detect the vibration peak of MSNs with different surface modification, including MSN, MSN-SH, MSN-SS-COOH, MSN/Res-COOH and MSN/Res-PEI-FA; C, UV spectrophotometry was used to detect the characteristic absorption peak of MSN with different surface modification, including MSN, MSN-SH, MSN-SS-COOH, MSN/Res-COOH and MSN/Res-PEI-FA.

### 3.2 Cytotoxicity assay of Res-loaded MSN

**Figure 2.**
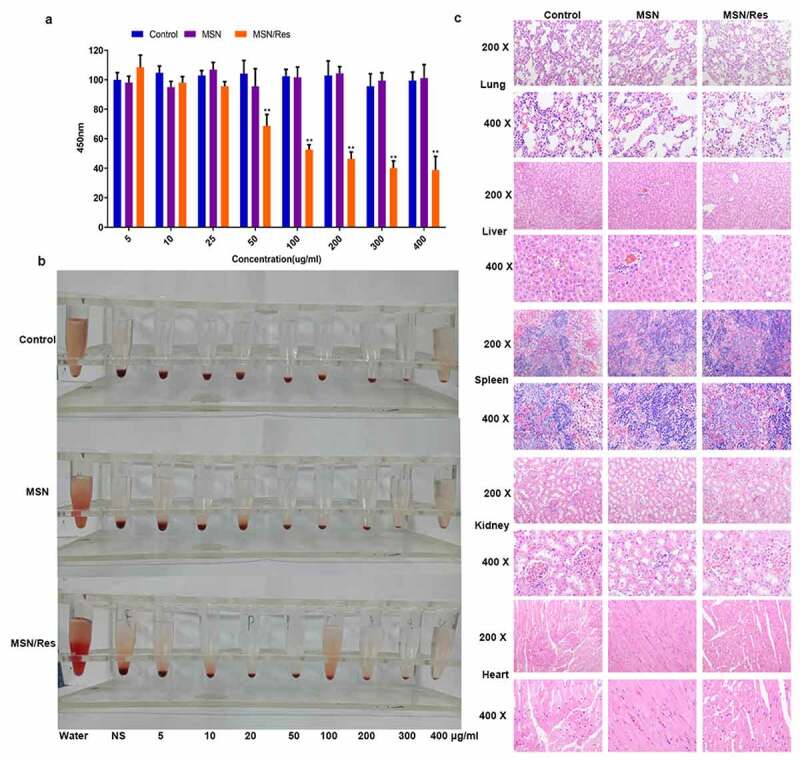
Cytotoxicity assay of Res-loaded MSN

The cytotoxicity of MSNs plays a crucial role in assessing whether they could be used for drug delivery. CCK-8 analysis showed that the inhibition rate of MSN-Res on cells reached approximately 50% at 100 μg/ml concentration, while the carrier nanomaterials were very toxic to cells (Figure 2a). The hemolysis ratio of all formulations was <5%, indicating good hemocompatibility of MSN (Figure 2b). In addition, in vivo safety evaluation indicated that compared with the control group, no injury to the heart, liver, spleen, lung, or kidney was observed after MSN-SH or MSN-Res treatment (Figure 2c).

A, Cell proliferation affected by MSN or Res-loaded MSNs with different concentrations from 0 to 400 μg/mL. Data shown as mean±SD. **P < 0.01, MSN/Res group Vs Control group; B, Hemolysis rate affected by MSN or Res-loaded MSNs with different concentrations from 0 to 400 μg/mL; C, Hematoxylin and eosin (H&E) staining was used to determine the presence of injury in the heart, liver, spleen, lung and kidney after MSN or Res-loaded MSN treatment. Magnification, 200× and 400 × .

### 3.3 Res-loaded MSN showed better suppressive effect on HGC-27 and AGS cells than treatment with Res in vitro

**Figure 3.**
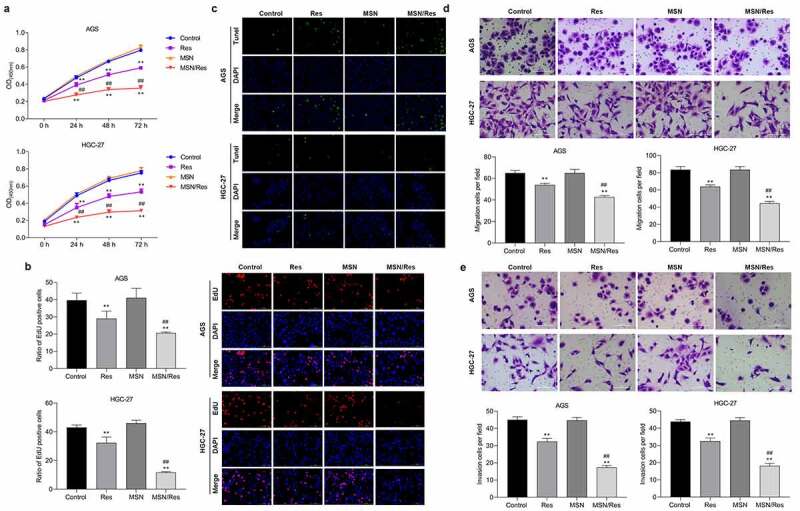
Res-loaded MSN showed better suppressive effect on HGC-27 and AGS cells than treatment with Res in vitro

In order to determine the effect of Res-loaded MSN on gastric cancer, the proliferation, migration, invasion, and apoptosis of HGC-27 and AGS cells were detected. CCK-8 analysis showed that the proliferation of both HGC-27 and AGS cells was dramatically inhibited by treatment with Res or Res-loaded MSNs (Figure 3a). EdU analysis also confirmed these results. The proliferation of HGC-27 and AGS cells was dramatically reduced when treated with Res or Res-loaded MSN compared with the control group (Figure 3b). TUNEL analysis showed that the number of apoptotic HGC-27 and AGS cells was significantly elevated when treated with Res or Res-loaded MSN compared with the control group (Figure 3c). Furthermore, we found that migration and invasion were greatly inhibited by treatment with Res or Res-loaded MSNs compared with the control group (Figure 3d and E). In addition, we found that Res-loaded MSN could further inhibit the proliferation, migration, and invasion, while promoting apoptosis in HGC-27 and AGS cells compared to treatment with Res (Figure 3a-E). These results demonstrated that Res-loaded MSNs had a better treatment effect on gastric cancer than direct Res treatment in vitro.

A, CCK-8 analysis was used to detect the proliferation in both HGC-27 and AGS cells after treatment with Res, MSN-SH or Res-loaded MSN for 24, 48 and 72 h; B, HGC-27 and AGS cell proliferation after treatment with Res, MSN-SH or Res-loaded MSN was confirmed by EdU assay. Scale bar = 100 μm; C, Apoptosis after treatment with Res, MSN-SH or Res-loaded MSN was detected by terminal deoxynucleotidyl transferase biotin-dUTP nick end labeling (TUNEL). Scale Bar = 100 μm; D and E, Migration and invasion after Res, MSN-SH or Res-loaded MSN treatment was detected by Transwell analysis. Scale Bar = 100 μm; Data shown as mean±SD. **, P < 0.01, Res group Vs MSN-SH or Control group; ##, P < 0.01, MSN/Res group Vs Res group.

### 3.4 Res-loaded MSN showed better anticancer effect on gastric cancer than treatment with Res in vivo

**Figure 4.**
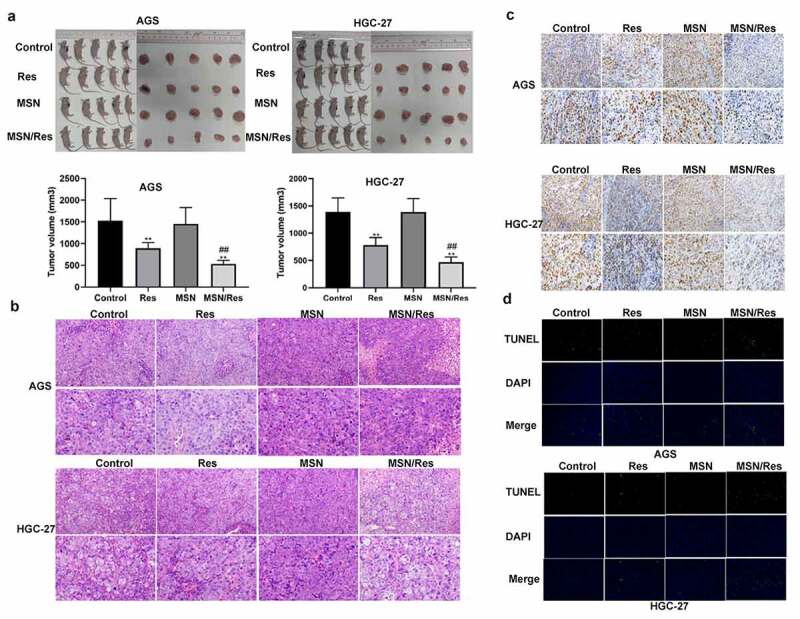
Res-loaded MSN showed better anticancer effect on gastric cancer than treatment with Res in vivo

To further confirm the effect of Res-loaded MSN in gastric cancer, a tumor-bearing nude mouse model with HGC-27 and AGS cells was established. Tumor size analysis showed that a greater reduction in tumor size was observed following treatment with Res-loaded MSN compared to those treated with Res alone (Figure 4a). Moreover, H&E analysis showed that inflammatory cell infiltration was greatly alleviated when treated with Res-loaded MSN compared with the control and Res groups (Figure 4b). Ki67 immunohistochemical staining indicated that the proliferation of HGC-27 and AGS cells was significantly inhibited when treated with Res or Res-loaded MSN compared with the control group (Figure 4c). This result was further supported by the TUNEL assay results, which showed that the number of apoptotic HGC-27 and AGS cells was significantly elevated when treated with Res or Res-loaded MSN compared with the control group (Figure 4d). Furthermore, the proliferation of HGC-27 and AGS cells was further inhibited, while apoptosis was further promoted when treated with Res-loaded MSN compared with Res (Figure 4c and d). These results demonstrated that Res-loaded MSNs showed better anticancer effects on gastric cancer than treatment with Res alone in vivo.

A, Tumor size in tumor-bearing nude mouse model with HGC-27 and AGS cells treated with Res, MSN-SH, or Res-loaded MSNs; B, Inflammatory cell infiltration after treatment with Res, MSN-SH, or Res-loaded MSNs was detected by H&E staining. Magnification, 200× and 400×; C, The proliferation of HGC-27 and AGS cells in tumor-bearing nude mice was detected by Ki67 immunohistochemical staining. Magnification, 200× and 400×; D, Apoptotic HGC-27 and AGS cells treated with Res, MSH-SH, or Res-loaded MSN were detected by TUNEL. Scale bar = 100 μm; Data shown as mean±SD. **, P < 0.01, Res group Vs MSN-SH or Control group; ##, P < 0.01, MSN/Res group Vs Res group.

## 4. Discussion

Res has shown great advantages in the treatment of gastric cancer [22]. Since numerous signaling pathways are involved in the dynamic progression of gastric cancer, the role of Res in regulating multiple molecular signaling pathways related to gastric cancer allows it to be a candidate member in combination therapy of gastric cancer, thus preventing tumor cells from developing resistance to chemotherapy [9]. However, due to its water solubility and low bioavailability, there are currently no clinical trials investigating the antitumor effect of Res on gastric cancer [8,23].

MSNs have high drug loading capacity, good biocompatibility, and easy surface modification. They also have tunable pore sizes, which allows for controlled drug release [24]. However, not much is known about Res-modified MSNs, especially in gastric cancer. In the present study, we successfully prepared Res-modified MSNs and characterized their characteristics. Functional analysis indicated that Res-modified MSNs presented better anti-tumor effects in the treatment of gastric cancer than Res alone.

Gastric cancer is one of the most common malignant tumors worldwide, with high morbidity and mortality [25]. Chemotherapy is an important treatment method for gastric cancer. However, currently used chemotherapy drugs still lack ideal therapeutic effects and have large toxic side effects [26]. Res is a natural polyphenol compound [27] that has been reported to have anti-tumor effects through anti-oxidation, regulation of cell redox metabolism, cell autophagy interference, reduction of cell invasiveness, inhibition of tumor cell proliferation, and induction of tumor cell apoptosis [28,29]. A large number of studies have also shown that Res plays crucial roles in the treatment of gastric cancer [10]. Xu et al. indicated that Res counteracted hypoxia-induced gastric cancer invasion and EMT by suppressing the hedgehog pathway [30]. Yang et al. demonstrated that Res inhibited the invasion and migration of human gastric cancer cells by inhibiting MALAT1-mediated EMT transition [31]. Consistent with these findings, we found that Res inhibited proliferation, migration, and invasion while promoting apoptosis in HGC-27 and AGS cells, and greatly alleviated tumor size and inflammatory cell infiltration in vivo. These results demonstrate that Res exerts an anti-tumor effect on the development of gastric cancer. However, although Res has strong physiological activity, poor water solubility, easy oxidation and decomposition, its fast metabolism in the body and low oral bioavailability limit its clinical application and development [32]. Therefore, establishing an efficient Res delivery system, improving Res targeting, and enhancing Res bioavailability is particularly important.

In recent years, with the development of nanomedicine, nanomaterials have received widespread attention in the field of tumor treatment because they are effective carriers of chemotherapeutic drugs, which can target the delivery of drugs to the tumor and reduce the side effects of chemotherapeutic drugs. MSNs have the advantages of strong tumor targeting, good biocompatibility, low toxicity, and controlled drug release after mesoporous modification [17]. It has been widely used to load small-molecule chemotherapeutics, nucleic acids, proteins, and other biological macromolecules, and has been successfully applied to basic research on multimodal tumor therapy [33,34], as well as in gastric cancer. For example, quercetin and doxorubicin co-delivery using MSN enhanced the efficacy of gastric carcinoma chemotherapy in an SGC7901/ADR tumor-bearing mouse model [18], and the combination of anti-miR21 and RSV in a targeted nanocarrier MSNS presented anticancer efficacy by promoting cell apoptosis, suggesting that MSNs might be a promising drug delivery system for gastric cancer therapy [19]. Moreover, since the surface of MSNs contains a large number of silanol groups, it is easy to functionalize, which could be utilized to control drug loading and drug release. In the present study, we successfully prepared Res-loaded MSNs containing PEI and FA, which had a characteristic absorption peak at 318 nm with high biosafety. The anti-tumor effect of Res-loaded MSN demonstrated that the Res-loaded MSN showed better anticancer effect than treatment with Res alone on gastric cancer therapy both in vitro and in vivo. These results demonstrate that MSNS is a promising active delivery system for Res, which has potential value for future applications.

## 5. Conclusion

In summary, we demonstrated the feasibility of the application of the MSN-Res drug delivery system in gastric cancer treatment, and successfully loaded functionalized MSNs with Res, which adsorbed on the cell surface without destroying its membrane or cell morphology and showed high biological safety. To some extent, these findings contribute to facilitating the process of clinical translation. Further analysis indicated that MSNs with Res presented better anticancer effects on the development of gastric cancer than Res alone in vitro and in vivo, indicating that MSN-Res delivered by the MSN system could be a promising drug delivery system for the treatment of gastric cancer. Further studies with efficacy tests are warranted before clinical validation.

## Research highlights

- Resveratrol was successfully loaded onto modified Mesoporous silica nanoparticles.
- Resveratrol-loaded Mesoporous silica nanoparticles exhibited high biosafety.
- Res-loaded MSNs inhibited the proliferation, migration, invasion of tumor cells.
- Res-loaded MSNs promoted tumor cell apoptosis.

Res-loaded MSNs showed better suppressive effect on gastric cancer than Res
